# Genetic analysis shows low levels of hybridization between African wildcats (*Felis silvestris lybica*) and domestic cats (*F. s. catus*) in South Africa

**DOI:** 10.1002/ece3.1275

**Published:** 2014-12-23

**Authors:** Johannes J Le Roux, Llewellyn C Foxcroft, Marna Herbst, Sandra MacFadyen

**Affiliations:** 1 Centre for Invasion Biology, Department of Botany & Zoology, Stellenbosch UniversityMatieland, 7602, South Africa; 2 Conservation Services, South African National ParksSkukuza, 1350, South Africa; 3 Conservation Services, South African National ParksPhalaborwa, 1390, South Africa; 4 Department of Botany & Zoology, Stellenbosch UniversityMatieland, 7602, South Africa

**Keywords:** African wildcat, feral cats, genetic introgression, human population pressure, hybridization, population genetic structure

## Abstract

Hybridization between domestic and wild animals is a major concern for biodiversity conservation, and as habitats become increasingly fragmented, conserving biodiversity at all levels, including genetic, becomes increasingly important. Except for tropical forests and true deserts, African wildcats occur across the African continent; however, almost no work has been carried out to assess its genetic status and extent of hybridization with domestic cats. For example, in South Africa it has been argued that the long-term viability of maintaining pure wildcat populations lies in large protected areas only, isolated from human populations. Two of the largest protected areas in Africa, the Kgalagadi Transfrontier and Kruger National Parks, as well as the size of South Africa and range of landscape uses, provide a model situation to assess how habitat fragmentation and heterogeneity influences the genetic purity of African wildcats. Using population genetic and home range data, we examined the genetic purity of African wildcats and their suspected hybrids across South Africa, including areas within and outside of protected areas. Overall, we found African wildcat populations to be genetically relatively pure, but instances of hybridization and a significant relationship between the genetic distinctiveness (purity) of wildcats and human population pressure were evident. The genetically purest African wildcats were found in the Kgalagadi Transfrontier Park, while samples from around Kruger National Park showed cause for concern, especially combined with the substantial human population density along the park's boundary. While African wildcat populations in South Africa generally appear to be genetically pure, with low levels of hybridization, our genetic data do suggest that protected areas may play an important role in maintaining genetic purity by reducing the likelihood of contact with domestic cats. We suggest that approaches such as corridors between protected areas are unlikely to remain effective for wildcat conservation, as the proximity to human settlements around these areas is projected to increase the wild/domestic animal interface. Thus, large, isolated protected areas will become increasingly important for wildcat conservation and efforts need to be made to prevent introduction of domestic cats into these areas.

## Introduction

Despite international conservation interventions, global biodiversity continues to decline (Butchart et al. 2010). This necessitates an improved understanding of the factors that impact all levels of biodiversity, from genes, to populations, communities, and ecosystems (Sutherland et al. 2012). Given the challenges faced by conserving biodiversity globally, the role of protected areas will remain fundamentally important for future efforts (SCBD 2008; Hoffmann et al. 2010; Butchart et al. 2012). However, due to their often limited geographic ranges, many smaller protected areas are becoming increasingly susceptible to factors such as land use change and habitat loss (Maiorano et al. 2008), invasive alien species (e.g. Foxcroft et al. 2013), and climate change (Butchart et al. 2010, 2012). Moreover, the extent to which protected areas contribute to a single species’ conservation may also be highly taxon-dependent, being influenced by, for example, dispersal abilities and resource availability.

Protected areas generally aim to conserve as much natural habitat as possible, buffering the biodiversity, ecosystem services, and other benefits they accrue, against the various anthropogenic factors outside their boundaries (Geldmann et al. 2013). One such example is to prevent contact and subsequent interbreeding between wild populations and their closely related domestic counterparts, which may lead to introgressive hybridization (Macdonald et al. 1989; Allendorf et al. 2001). It has been suggested that hybridization is largely underappreciated as a conservation concern (Rhymer and Simberloff 1996), with some even considering the loss of genetically distinct populations within a species as comparable to the loss of an entire species (Ehrlich 1988). Such genetic “pollution” is commonplace and has been documented for many taxa, including mammals (e.g., wolves, Gotelli et al. 1994), birds (e.g., partridge, Barilani et al. 2007), fish (e.g., Atlantic salmon, Ayllon et al. 2006), plants (e.g., *Senecio* spp., Prentis et al. 2007), and invertebrates (e.g., *Leptocoris* soapberry bugs, Andres et al. 2013). Hybridization may lead to the replacement of wild populations and/or dramatic changes to the genetic makeup that evolved *in situ* and therefore, in the long term, negatively impacts evolutionary potential and species diversity (Rhymer and Simberloff 1996; Allendorf et al. 2001). Some authors have argued that hybridization between previously isolated populations can act as a source of adaptive genetic variation, especially when these populations experience temporary fitness declines, for example, invasive species undergoing a bottleneck (Verhoeven et al. 2010). However, many authors have argued that hybridization between domestic taxa and their wild relatives leads to outbreeding depression and reduced fitness, resulting in the loss of local adaptations rather than increased adaptability (e.g., Orr 1998, Seehausen 2004).

**Figure 1:**
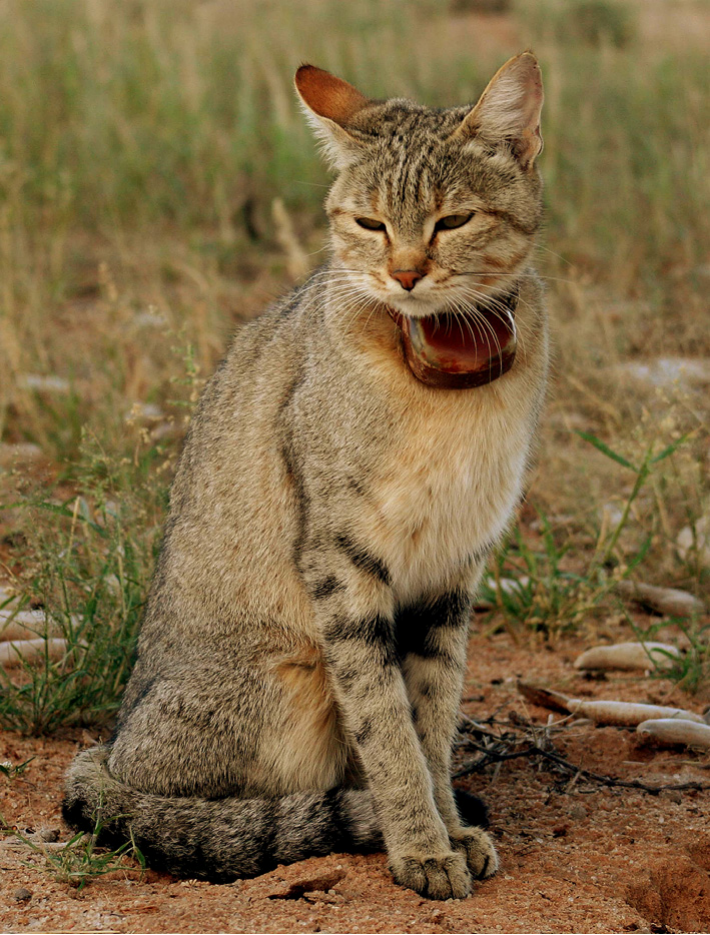
African wildcat (*Felis silvestris lybica*) in Kgalagadi Transfrontier Park (South Africa/Botswana) (Photo M. Herbst).

Hybridization is especially common between intraspecific entities, such as subspecies, due to incomplete reproductive isolation and therefore a higher likelihood of successful interbreeding (Rhymer and Simberloff 1996; Levin 2002; Randi 2008). For example, the recent divergence (∼ 9000 years ago) between subspecies of domestic cats (*Felis silvestris catus*) and their wild ancestors suggests little or no reproductive barriers may exist (Driscoll 2007). Indeed, reports on the genetic purity of European wildcat populations (*F. s. silvestris*) confirm high levels of admixture with domestic cats in Hungary and Scotland (Beaumont et al. 2001; Daniels et al. 2001; Pierpaoli et al. 2003; Lecis et al. 2006; Randi 2008). Curiously, it has also been shown that wildcat populations from Italy, Germany, and Portugal appear genetically distinct, with low levels of interbreeding with domestic cats (Randi et al. 2001; Pierpaoli et al. 2003; Lecis et al. 2006; Oliveira et al. 2008a,b). None of these European studies explicitly stated whether any of these wildcat populations originated from relatively isolated parts within the species’ distribution ranges or even protected areas. African wildcat (*F. s. lybica*, Fig.1) populations from southern Africa still appeared genetically distinct and pure before 2000 (Wiseman et al. 2000). This is despite the general concern that hybridization with domestic cats might be occurring extensively, to the point where it was generally accepted that “hybridization will lead to the virtual extinction of the African wildcat as we know it at present” (Smithers 1986). However, the analysis by Wiseman et al. (2000) only included a limited number of samples - 16 wildcats obtained over a large geographical range and mostly from isolated rural areas -and may therefore not have accurately captured all the genetic diversity or the incidence and extent of hybridization in South Africa.

Here, we aim to assess the genetic status of African wildcat populations across South Africa in various ways. First, we wanted to examine the overall genetic purity of African wildcat populations in South Africa. We then wanted to determine whether the genetic purity of African wildcats is influenced by their proximity to protected areas, and lastly, taking into account the home ranges of African wildcats, we wanted to infer the impacts of spatial proximity of African wildcats to human influences on their genetic purity.

## Materials and Methods

### Animal collections and DNA extraction

**Figure 2:**
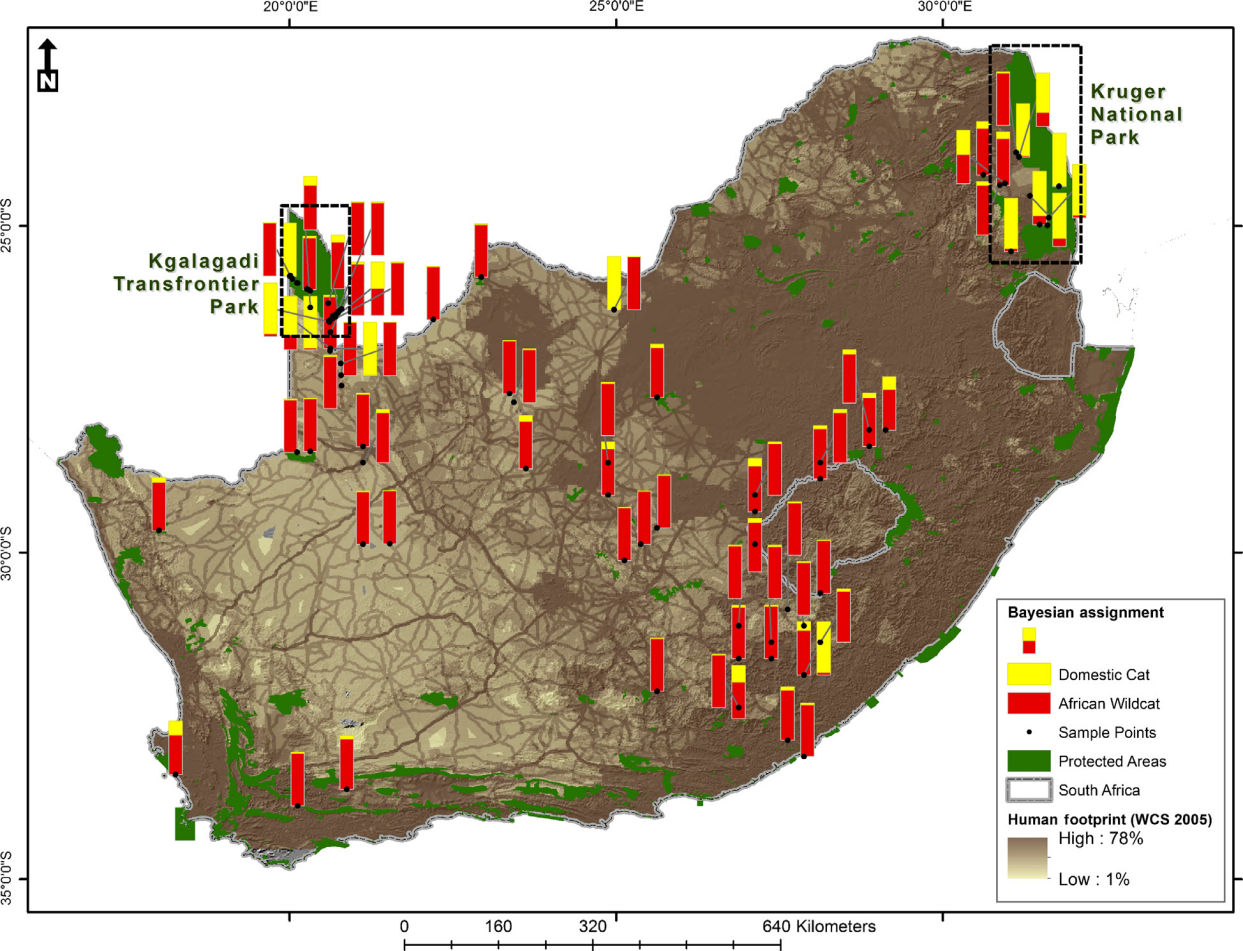
Distribution of collection sites of cats included in this study across South Africa in relation to formal protected areas and human footprint pressure. The proportion Bayesian assignments based 13 microsatellites of sampled localities to the African wildcat (*q*_AWC_) and domestic cat (*q*_DC_) genetic groups are indicated as bar graphs. As African wildcat genetic purity (proportion Bayesian assignment to the African wildcat cluster) increases, there is an associated decrease in the global human footprint influence index (*P *=* *0.0003) and an increase in the distance to the nearest town (*P *=* *0.026).

Tissue and hair material of African wildcats and domestic cats were obtained from numerous sources (Table S1); first, historical collections (*n *=* *46) were obtained as dried tissue material from various museums throughout South Africa (Fig.2). In addition, contemporary collections were donated by private conservation agencies and landowners (*n *=* *13). Due to the importance of Kgalagadi Transfrontier Park (KTP) as a large protected area supporting a high African wildcat population, 47 samples were collected within the KTP, and 10 samples outside KTP, either as road kill or by trapping, between April 2003 and December 2006 (Herbst 2009). The latter tissue samples were preserved in 95% ethanol and hair samples, containing follicles, in plastic bags. Lastly, 49 domestic cat samples were obtained from the University of Pretoria's Veterinary Genetics Laboratory (Herbst 2009), private veterinarians in Cape Town and the Animal Welfare Society in Stellenbosch, South Africa.

Initial classification of cats into African wildcat and putative hybrid classes was based on the following morphological characteristics: African wildcats have distinctive coat-patterns, characteristic long legs, and a prominent reddish tint behind the ears (Fig.1, Smithers 1983). Cats with typical wildcat markings and coloration but with darker ears were classified as putative hybrids. Additionally, the legs of hybrids cats are visibly shorter than those of pure wildcats. Wildcats were also classified as putative hybrids when they were kept as domestic pets and when the owners suspected or knew that they were cross-bred. In total, we obtained 165 tissue and hair samples, including 116 putative African wildcats or their suspected hybrids, and 49 domestic cats.

DNA from tissue material was extracted using the Cell Lysis stock solution (10 mmol/L Tris–HCl pH 8.0, 50 mmol/L NaCl, 10 mmol/L EDTA) and phenol–chloroform–isoamylalcohol (Sigma-Aldrich). DNA from hair samples was extracted with 200 mmol/L NaOH and 200 mmol/L HCl, 100 mmol/L Tris–HCl, pH 8.5 (Herbst 2009). DNA from desiccated museum samples was extracted according to the manufacturer's protocol with the Qiagen DNA Tissue kit (Qiagen, supplied by WhiteHead Scientific, Cape Town, South Africa). All DNA samples were quantified using a micro-volume UV-Vis spectrophotometer (Nanodrop, Thermo Fisher Scienti-fic, Wilmington, MA) and good quality genomic DNA (A_260/280_ = 1.8 and A_260/230_ = 2.0) diluted to a final concentration of 20 ng/*μ*L and stored at −80°C.

### Microsatellite genotyping

We selected 13 unlinked microsatellite markers previously characterized in domestic cats (Menotti-Raymond et al. 1999). Forward primers for all loci were fluorescently labeled and PCRs optimized into three multiplexes (see Table S2 for details). Briefly, each PCR contained about 20 ng of genomic DNA, 0.2 U Taq DNA polymerase (Kapa Biosystems, supplied by Lasec, Cape Town, South Africa), 1 X PCR reaction buffer, 0.5 mm MgCl_2_, primers at specific concentrations (Table S2), with the final reaction volume adjusted to 10 *μ*L with distilled water. All multiplex reactions were amplified using the following thermal cycle: an initial denaturation at 95°C for 3 min, followed by 30 cycles of initial denaturation at 95°C for 15 s, annealing at 60°C for 30 s, and elongation at 72°C for 30 s. A final extension was carried out at 72°C for 15 min. Successful amplification was verified using agarose gel electrophoresis. Purified PCR fragments were separated on an ABI Prism 3100 Genetic Analyzer (Applied Biosystems, Foster City, CA), using GENESCAN™-500 (-250) as an internal size standard (Appl-ied Biosystems). Allele sizes were visualized and scored using GENEMARKER v1.95 (SoftGenetics LLC, State College, PA).

### Genetic structure and purity of African wildcats in South Africa

Overall population genetic structure (for all individuals) was estimated using Bayesian assignment tests implemented in STRUCTURE v2.3.4 (Pritchard et al. 2000). STRUCTURE uses Bayesian Monte–Carlo Markov chain sampling to identify the optimal number of genetic clusters for a given dataset by reducing departures from Hardy–Weinberg and linkage equilibrium expectations within genetic clusters. We tested for *K *=* *2–8 (number of genetic clusters) and ran five independent models for each value of *K*. Each model consisted of 1,000,000 generations of which the first 100,000 were discarded as burn-in. We also applied the admixture model with correlated allele frequencies as we suspected hybrid individuals to be present in the dataset based on morphological observations. The optimal *K* value was determined using the method described by Evanno et al. (2005) and STRUCTURE Harvester (Earl and von Holdt 2012). A principal component analysis (PCA) was also conducted to visualize the genetic structure among groups using the *adegenet* package in the R statistical environment (Jombart et al. 2008; R Core Development 2010).

STRUCTURE calculates assignment values as the proportion (*q*_*ik*_) of each individual's multilocus genotype (*I*) derived from each of the predefined *K* number of clusters. Therefore, while individual genotypes may show membership to more than one cluster (i.e., being admixed), the sum of its *q*_*ik*_ is always one. These assignment values can therefore be useful in the identification of hybrid individuals. However, the proportion of pure and admixed (hybrid) individuals within a given sample will be strongly influenced by the validity of the assumed priors and the efficiency of analyzed loci used in the Bayesian analysis, and cannot be statistically tested (Oliveira et al. 2008a,b). Thus, to validate the identification of pure parental (domestic and wildcats) and admixed individuals identified in the STRUCTURE analysis, we also created and analyzed a simulated genotype dataset (e.g., see O'Brien et al. 2009). To simulate different datasets, we selected two subsamples from our data consisting of the 30 individuals that had *q*_*ik*_* ≥* 0.98 for the “African wildcat” and “domestic cat” clusters, respectively. This threshold is very conservative compared to similar analyses used in other studies on wildcats (e.g., see Pierpaoli et al. 2003; Lecis et al. 2006; Oliveira et al. 2008a,b). The function *hybridize* in the R package *adegenet* (Jombart et al. 2008; R Development Core Team 2010) was used to simulate six different genotype datasets each consisting of 100 genotypes of: pure wildcats, pure domestic cats, F1 hybrids, F2 hybrids, and F1 hybrids backcrossed with African wildcats and F1 hybrids backcrossed with domestic cats. Simulated genotypes were analyzed with the same parameter and prior settings used for the full collected dataset described above, but constraining *K* to two clusters (see Results for actual data). 95% CI intervals for *q*_*ik*_ – values obtained from simulated genotypes were determined in R (R Core Development Team 2010) for each scenario to assess the efficiency of the admixture analysis to detect the different classes of F1, F2, and backcrossed genotypes in our data.

### Dispersion of genetic purity in relation to protected areas

We first assigned all individual cats as wildcat, domestic cat, or admixed (see above). Using these assignment values and their standard deviations, we were able to class individual genotypes as genetically pure (African wildcat or domestic cat), F1, F2, or F1 backcrossed (Table S1). We also calculated the distance of all sample sites from the boundaries of formally protected areas as described by SANBI (2011). Sites between 0–5 km of a protected area were labeled “inside”, while sites >5 km were labeled “outside” protected areas. All pure domestic cat and F1 cats backcrossed with domestic cat individuals were excluded from this analysis. A box-and-whisker plot of the Bayesian assignment values (to the African wildcat cluster) was used to illustrate the dispersion of genetic assignment values inside and outside protected areas. The significance of differences was determined by Kruskal–Wallis rank sum test using R statistical environment (R Core Development 2010).

### African wildcat home range

African wildcat home range sizes were assessed in the southern region of the Kgalagadi Transfrontier Park. Eight African wildcats (three female and five male) were radio collared from 2003 to 2006 (46 months) (see Table S3 for more details). Home ranges were calculated using minimum convex polygons (MCP) (Mohr 1947), and overlap in home range was determined from 100% MCP estimates. MCP are considered a robust, nonparametric analysis of home range size when more than 30 independent points are available (Kenward and Hodder 1996), but are sensitive to outliers (Harris et al. 1990). Points from continuous observations of habituated individuals are temporally autocorrelated and this may result in an underestimation of home range size (Swihart and Slade 1985a,b). As African wildcats do not have a fixed den site but rest in different places each day, the resting positions can be considered biologically independent locations as they are separated by a period of differential activity (Minta 1992; Creel and Creel 2002). Home range data and spatial organization of wildcats *(F. silvestris*) are limited to short-term studies, small sample sizes, and opportunistic observations (Nowell and Jackson 1996). Although home range sizes show large variability, which could be due to varying densities, distribution of prey, and environmental conditions (Liberg and Sandell 1988; Adams 2001), our wildcat range estimates fall within the ranges of previous wildcat studies (Herbst 2009).

Two types of data were collected: (1) radiolocation observations, when only a radio-fix of the animal was recorded, and (2) continuous observations, when radio collared African wildcats were followed by a vehicle for varying periods of 1–14 h (an average of 6.0 ± 3.4 h for males and 4.7 ± 3.7 h for females). A rotation system was followed in order to obtain equal observation records for all cats. Over the course of the study 1538 h were spent with habituated wildcats (females = 881 h [*n *=* *3] and males = 657 h [*n *=* *5]).

### Influence of human population

The proximity of wild-collected cats (*n *=* *146) to human infrastructure and settlement density was assessed using the Global Human Footprint (GHF) Dataset of the Last of the Wild Project (WCS 2005) and National Geo-Spatial Information (NGI) of Populated Places (POP) in South Africa (www.ngi.gov.za, Fig.2). Sample locations were plotted in ArcMap 10.1 Geographic Information System (GIS) Software (ESRI, Redlands, CA, USA) from coordinates provided by museums and individual collectors. Using the results from home range estimates (see above), the points were buffered by a 15 km radius to simulate maximum potential home range area. Zonal statistics were calculated within these areas for GHF values and POP density and distance measures. The relationship between the genetic purity of African wildcat (proportion Bayesian assignment to the African wildcat [AWC] cluster) and different levels of human influence (GHF: standard deviation, majority value, minimum value, maximum value, mean; POP Density: mean, maximum value, minimum value, maximum value; and POP Distance to the nearest town was assessed using General Linear Models (GLM) in R (R Development Core Team 2010).

## Results

### Admixture and simulation analysis

**Figure 3:**
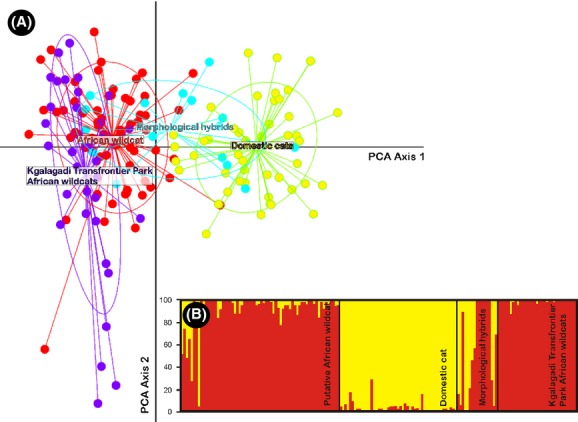
(A) Scatter plot of Principle Components Analysis showing the genetic structure between putative African wildcats (red), African wildcats from the Kgalagadi Transfrontier Park (purple), domestic cats (yellow), and morphological hybrids (light blue). (B) STRUCTURE bar plots where vertical axes illustrate the proportional assignment of individual genomes to the inferred genetic groups (*K *=* *2) for African wildcats outside protected areas, domestic cats, morphological hybrids, and individuals from the Kgalagadi Transfrontier Park. Membership of each individual's genome (*q*_AWC/DC_) to the two identified genetic clusters is indicated by different colors of vertical bars (red, African wildcat; yellow, domestic cat).

Bayesian assignment tests indicated that two genetic clusters exist, overall corresponding to African wildcats and domestic cats (Fig.3). Higher values of *K* were congruent with this finding, indicating that genetic clustering were mainly driven by differences between African wildcats and domestic cats (Fig. S1). Cats from the Kgalagadi Transfrontier Park assigned highly to the African wildcat genetic cluster (mean *q*_AWC_ = 0.982). Surprisingly, based on morphology, seven suspected hybrids assigned very highly to the African wildcat genetic cluster (*q*_AWC_ ≥ 0.89) and four to the domestic cat genetic cluster (*q*_DC_ ≥ 0.85) (Fig.2, Table1). All remaining morphological hybrids showed some level of admixture. Among individuals classified as domestic cats only one cat showed some sign of admixture (*q*_AWC_* =* 0.29). Of the putative African wildcats collected outside protected areas (*n *=* *68), seven individuals had admixed genotypes (*q*_DC_: 0.23–0.79) while two cats assigned highly to the domestic cat cluster (*q*_DC_ ≥ 0.94). The STRUCTURE results were supported by scatter plots in the PCA that separated genetic groups (Fig.3); however, axis 1 and 2 only explained 12.5% of the variation in the data.

**Table 1:** Mean Bayesian assignment values to African wildcat genetic cluster (*q*_AWC_) and their 95% CI for pure parental individuals, F1 and F2 hybrids and backcrosses with wildcat and domestic cat populations based on simulated genotypes. Actual assignment values of putative hybrids and some pure wildcats collected in this study are given and their relation to simulated data indicated by asterisks

	AWC	F1	F2	AWC-backcross	DC-backcross	DC
Mean *q*_AWC_	0.932	0.515	0.510	0.759	0.264	0.070
95% CIs	0.928–0.935	0.509–0.521	0.498–0.524	0.750–0.768	0.255–0.271	0.067–0.073
Suspected hybrids						
C016 (*q*_AWC_: 0.154)						^*^
C023 (*q*_AWC_: 0.057)						^*^
C042 (*q*_AWC_: 0.888)	^*^					
C043 (*q*_AWC_: 0.008)						^*^
C107 (*q*_AWC_: 0.012)						^*^
C130 (*q*_AWC_: 0.200)					^*^	
C131 (*q*_AWC_: 0.453)			^*^			
C132 (*q*_AWC_: 0.555)		^*^				
C133 (*q*_AWC_: 0.991)	^*^					
C135 (*q*_AWC_: 0.990)	^*^					
C136 (*q*_AWC_: 0.989)	^*^					
C137 (*q*_AWC_: 0.981)	^*^					
C138 (*q*_AWC_: 0.980)	^*^					
C139 (*q*_AWC_: 0.889)	^*^					
C140 (*q*_AWC_: 0.275)					^*^	
C168 (*q*_AWC_: 0.048)						^*^
C183 (*q*_AWC_: 0.689)				^*^		
Putative pure wildcats						
C003 (*q*_AWC_: 0.514)		^*^				
C006 (*q*_AWC_: 0.739)				^*^		
C008 (*q*_AWC_: 0.474)			^*^			
C009 (*q*_AWC_: 0.643)		^*^				
C011 (*q*_AWC_: 0.260)					^*^	
C027 (*q*_AWC_: 0.042)						^*^
C151 (*q*_AWC_: 0.766)				^*^		

**Figure 4:**
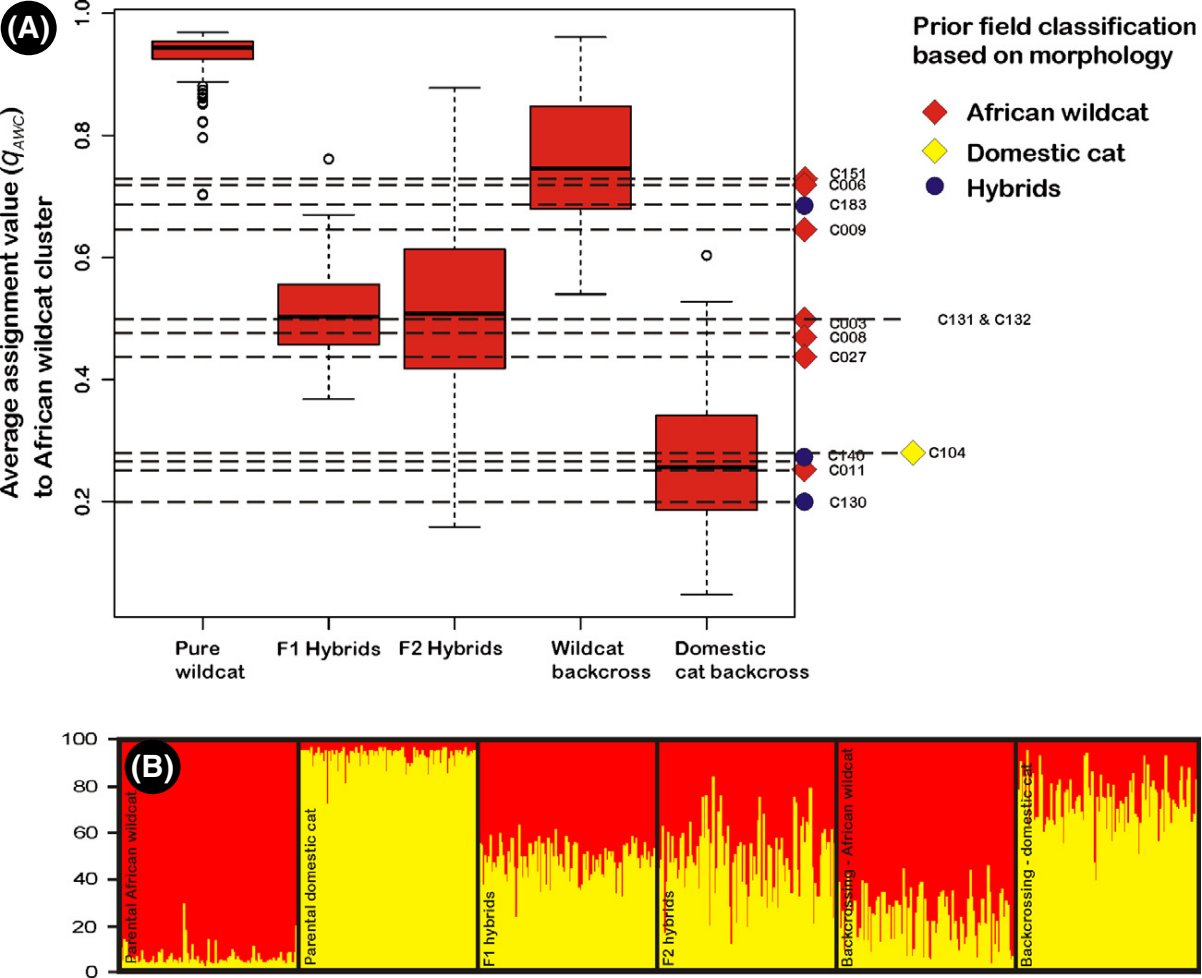
(A) Boxplots illustrating the variation in assignment probabilities to the African wildcat genetic cluster (*q*_AWC_) based on simulated genotypes for pure parental African wildcat (AWC), F1, F2, and backcrossed individuals identified in STRUCTURE. Dashed lines indicate actual *q*_AWC_ –values of admixed individuals obtained from our field-collected data that did not assign with high probability (*q*_AWC__/DC_ ≥0.8) to any of the parental genetic clusters. (B) STRUCTURE bar plots of simulated pure parental, F1, F2, and backcrossed genotypes (100 each). Membership of each individual's genome (*q*_AWC__/DC_) to the two genetic clusters (domestic, yellow and African wildcat, red) is indicated by vertical bars.

At a probabilistic assignment threshold of *q*_AWC/DC_ = 0.80 (see Pierpaoli et al. 2003; Lecis et al. 2006; Oliveira et al. 2008a,b), our simulation results indicated that the admixture analysis was able to efficiently recognize 99% of pure parental individuals (Fig.4, Table1). Similarly, all F1 hybrids were correctly identified as admixed cats with the highest assignment to African wildcat and domestic cat clusters being *q*_AWC_ = 0.76 and *q*_DC_ = 0.631, respectively. However, 4% of F2 individuals assigned to one of the parental clusters with *q*_AWC/DC_ ≥0.8. Simulation results also indicated that it is problematic to distinguish F1 and F2 individuals from one another. In total, 30.5% of all backcrossed individuals had *q* values ≥0.8 to one of the parental genetic clusters, indicating that distinguishing pure cats from backcrossed individuals might also be problematic in some instances (Fig.4). Within this framework, we were able to validate the admixture results from our field-collected data. Some suspected hybrids (*n *=* *6) represent pure domestic (*n *=* *5) and wildcats (*n *=* *1), while other putative wildcats (*n *=* *7) are hybrid and possibly backcrossed individuals (Table1, Fig.4). Moreover, African wildcats from within protected areas were genetically pure (Fig.3).

### Genetic dispersion

**Figure 5:**
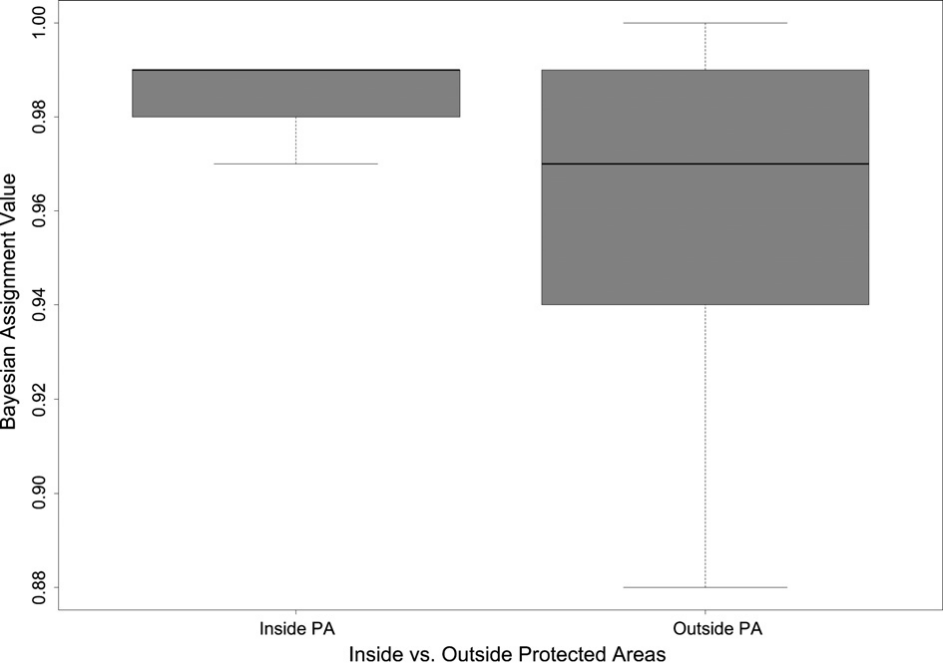
Box-and-whisker plot illustrating levels of genetic dispersion inside and outside protected areas. All putative wildcat samples collected between 0 and 5 km of a protected area were classified as “inside” (*n *= 47) and samples >5 km were classified as “outside” (*n *= 53) protected areas. Bayesian assignment values (to AWC genetic cluster) were significantly less dispersed for African wildcats inside protected areas, compared to those from outside protected areas (Kruskal–Wallis χ^2^ = 5.2705, *P *=* *0.02169, df = 99).

Levels of genetic dispersion were significantly lower for African wildcats inside or within 5 km of protected areas and showed higher genetic purity (assignment to AWC cluster), compared to wildcats from outside protected areas (Kruskal–Wallis χ^2^ = 5.2705, *P *=* *0.02169, df = 99) (Fig.5).

### Home range patterns of African wildcats in KTP

Annual home range estimates (MCP 95%) for adult males was 7.7 (±3.5) km^2^ and 3.5 (±1.0) km^2^ in adult female African wildcats. The annual home range sizes (95% MCP) of adult male cats were significantly larger (between 1.6 and 2.2 times) than female wildcats (Mann–Whitney *U*-test, *Z* = 2.3, *P *<* *0.02, df = 7). Female cats displayed extensive overlap of home ranges (average of 33.4 ± 13.4%), while the overlap between male home ranges was limited (average of 3.5 ± 5.3%), with no overlap of core areas. Males overlapped extensively with the home ranges of up to four females.

### Influence of human footprint on wildcat genetic purity

Significant relationships were observed between Bayesian assignment values to the AWC genetic cluster and the maximum GHF value of each potential home range for each sample (*P *=* *0.0003), standard deviation within zones (*P *=* *0.0097), and distance to the nearest town (*P *=* *0.026; Table2). This suggests that as AWC genetic purity increases there is an associated decrease in the GHF human influence index and an increase in the distance to the nearest town (see Fig.2).

**Table 2:** Generalized linear model results of proportion African wildcat Bayesian assignment value to human footprint and distance to human settlements.

	Estimate	SE	*t*- value	Significance
Intercept	0.693412	0.184365	3.761	***0.000251
HFSTD	−0.075179	0.028671	−2.622	**0.009743
HFMAJ	−0.017135	0.011679	−1.467	0.144662
HFMIN	−0.017155	0.011066	−1.550	0.123438
HFMAX	0.019323	0.005240	3.688	***0.000327
HFMEAN	−0.006083	0.015064	−0.404	0.686984
denTwnMEAN	0.012292	0.017438	0.705	0.482084
denTwnMAX	0.008498	0.027008	0.315	0.753507
disTwnMIN	0.004681	0.010306	0.454	0.650448
disTwnMAX	−0.035246	0.024971	−1.411	0.160406
distTown	1.491595	0.663428	2.248	*0.026177

HFSTD – Global Human Footprint (GHF) standard deviation; HFMAJ – GHF majority value; HFMIN – GHF minimum value; HFMAX – GHF maximum value; HFMEAN – GHF mean; denTwnMEAN – Populated Places in South Africa (POP) Density mean; denTwnMAX – POP Density maximum value; disTwnMIN – POP Distance minimum value; disTwnMAX – POP Distance maximum value; distTown – POP Distance to the nearest town.

*** Significant at *P *<* *0.001;

** Significant at *P *<* *0.01;

* Significant at *P *<* *0.05.

## Discussion

Our genetic analyses of African wildcat collections spanning five decades, and from regions throughout South Africa, indicate high genetic distinctiveness from their domestic counterparts, with seemingly limited hybridization and introgression. This finding is maybe surprising given the genetic status of wildcats elsewhere in the world (e.g., Beaumont et al. 2001; Daniels et al. 2001; Pierpaoli et al. 2003; Lecis et al. 2006), and that feral cat populations are of growing concern in South Africa due to their presence across the entire African wildcat range, and in particular in urban and suburban conservancies (Smithers 1986; Tennent and Downs 2008; Tennent et al. 2009). This has led to the general belief that hybridization with domestic cats might be the single most important long-term conservation threat to African wildcats in southern Africa (Smithers 1986; Nowell and Jackson 1996). Our results are also in agreement with those of Wiseman et al. (2000), who based their inferences on a limited sample size of African wildcats (*n *=* *16) and genetic diversity and structure, rather than admixture analysis.

Despite the genetic distinctiveness of African wildcat populations throughout South Africa, we identified a few genetic anomalies to this overall pattern. First, we identified seven putative wildcat specimens that showed varying levels of admixture, most likely resembling F1 or F2 hybrids and possibly backcrosses (Table1). All these specimens were collected close to urban environments. Moreover, some of these specimens were collected and donated by interested parties; often private individuals who may lack the taxonomic expertise to correctly identify pure African wildcat individuals based on morphology. Furthermore, some of these admixed cats had high genome assignments to the African wildcat cluster (*q*_AWC_ ≥0.643) indicating possible F2 offspring or even wildcat backcrosses, which may obscure morphological features distinguishing hybrids from parental phenotypes. This may also present problems where well-meaning members of public keep “wildcats” for breeding and reintroduction into the wild, or as pets in areas where wildcats are present, by increasing the chances for contact and breeding with wild populations.

It has been suggested that one of the most important contributions of protected areas to cat conservation is preventing hybridization from occurring (Nowell and Jackson 1996). However, due to landscape fragmentation and habitat loss, the size of protected areas is likely to become important in maintaining the ability of protected areas to continue this function (Nowell and Jackson 1996). The European wildcat is considered to be near-threatened in 25 member states of the European Union (Temple and Terry 2007) due to human persecution and habitat loss, including in protected areas (e.g., Doñana National Park, south-western Spain, Soto and Palomares 2013). Additionally, while some European wildcat and domestic cat populations still appear genetically distinct, contrasting patterns of genetic admixture have been identified, from recent and frequently hybridizing populations in Scotland and Hungary (Beaumont et al. 2001; Daniels et al. 2001; Pierpaoli et al. 2003; Lecis et al. 2006), to relatively low genetic introgression in populations in Italy, Germany, and Portugal (Randi et al. 2001; Pierpaoli et al. 2003; Lecis et al. 2006; Oliveira et al. 2008b; Randi 2008). While the African wildcat is not a protected species in southern Africa (listed as “Least concern” in the National Red List status (2004) and Global Red List status (2008), and also proposed as “Least Concern” in the upcoming (2014) National Red list), Nowell and Jackson (1996) suggested that the only long-term protection against introgression with domestic cats is in large isolated protected areas. However, many of South Africa's protected areas are found in close proximity to rapidly expanding urban areas and human settlements (Wittemyer et al. 2008). Not surprisingly, domestic cats have been recorded from 16 of South African National Parks’ 19 protected areas (Spear et al. 2011), and human population density surrounding a protected area has been shown to be a significant predictor of alien species richness in protected areas (Spear et al. 2013).

While the genetic status of wildcat populations in South Africa, including areas outside of protected areas, generally suggests minimal hybridization and introgression, the purest populations were found inside protected areas. Our results show that the African wildcat population in the southern KTP, one of Africa's larger conservation areas (∼3.6 million ha) is still genetically pure, with no signs of hybridization and introgression (but see Herbst 2009 for a single hybrid cat previously recorded in KTP), despite the occurrence of domestic cats on the southern periphery of the KTP. This is in contrast to the belief in the mid-1990s that no protected areas in South Africa were considered feasible for maintaining the genetic purity of African wildcats (Nowell and Jackson 1996), including the Kalahari Gemsbok National Park in South Africa, now part of the KTP. The closest human settlement is about 10 km away from the KTP boundary, with large livestock grazing farms bordering most of the park, normally associated with very low human population densities (Fig.2). It is plausible that at this distance and based on the home range estimates of African wildcats identified here, feral domestic cats associated with humans are less likely to come into contact with African wildcats within KTP. These results can therefore also be applied to the future conservation of African wildcat populations in protected areas, in suggesting minimum required buffer zones that would limit gene exchange, and by identifying areas of high risk for potential contact with feral cat populations. The northern parts of KTP are more isolated and thus the likelihood of the home ranges of wild and domestic cats overlapping can be assumed to be substantially lower than in our study area. Similar to KTP, one of South Africa's flagship protected areas, the Kruger National Park (KNP, ∼2 million ha), was excluded as being a long-term refuge for genetically pure African wildcats (Nowell and Jackson 1996). Due to the long, narrow shape of KNP, and a high human population density along the park's boundaries (Spear et al. 2013), African wildcat populations might be less isolated and thus more susceptible to contact with feral domestic cats.

Even when hybridization is prevalent and widespread within a species, it might be locally rare (Oliveira et al. 2008a). Reports in southern Africa predicted hybridization between African wildcats and domestic cats to be widespread (e.g., Smithers 1983), although at low levels (Wiseman et al. 2000). Our results indicate that the assumption of widespread hybridization is currently unwarranted, but needs to be managed in future. Moreover, our results emphasize the role protected areas play in maintaining the genetic integrity of wild populations and thus the conservation of regional biodiversity.

## Supporting Information

**Table S1.**Collection data for all cats included in this study, excluding domestic cats obtained from private veterinarians and donors. **Table S2.** Details of microsatellite primers used in this study. **Table S3.** Standard body measurements and mean annual home range for eight African wildcats observed during home range studies. **Figure S1.** STRUCTURE bar plots where vertical axes illustrate the proportional assignment of individual genomes to the inferred genetic groups (*K *=* *2–8) for African wildcats outside protected areas, domestic cats, morphological hybrids, and individuals from the Kgalagadi Transfrontier Park. Membership of each individual's genome to genetic clusters is indicated by different colors of vertical bars.
