# Supplementary material for: Genetic analysis shows low levels of hybridization between African wildcats (Felis silvestris lybica) and domestic cats (F. s. catus) in South Africa
Source: Ecol Evol. 2014 Dec 23;5(2):288–99. doi: 10.1002/ece3.1275 (PMC4314262; doi:10.1002/ece3.1275)
Supplement: Supplementary file 1 [file ece30005-0288-sd1.docx]

**Table S1: Collection data for cats included in this study, excluding domestic cats obtained from private veterinarians**

| **Sample** | **pDom** | | | **pAWC** | | **Processed** | | **Type** | | **ClassDescription** | | **finClass** | **origClass** | **DateColl** | **Mapsheet** | **Accuracy** | **LAT_Y** | **LONG_X** | **General location / Collector** |
| --- | --- | --- | --- | --- | --- | --- | --- | --- | --- | --- | --- | --- | --- | --- | --- | --- | --- | --- | --- |
| C003 | 0.49 | | | 0.51 | | Y | | Hair | | F1-2 HYB | | Hybrid | AWC | 2005 | 2430BD | GPS | -24.350029 | 30.954342 | Hoedspruit |
| C006 | 0.26 | | | 0.74 | | Y | | Hair | | F1 BX AWC | | AWC | AWC | 2011 | 3318AD | GPS | -33.396639 | 18.255583 | Jakkalsfontein Nature Reserve |
| C008 | 0.53 | | | 0.47 | | Y | | Hair | | F1-2 HYB | | Hybrid | AWC | 2011 | 2430BD | GPS | -24.350029 | 30.954342 | Hoedspruit |
| C009 | 0.36 | | | 0.64 | | Y | | Hair | | F1-2 HYB | | Hybrid | AWC | 2011 | 2430BD | GPS | -24.350029 | 30.954342 | Hoedspruit |
| C011 | 0.74 | | | 0.27 | | Y | | Hair | | F1 BX DC | | DC | AWC | 2010 | 2331CC | GPS | -23.943068 | 31.166270 | Phalaborwa, Kruger National Park |
| C016 | 0.85 | | | 0.16 | | Y | | Hair | | DC | | DC | Hybrid | 2010 | 2431CB | GPS | -24.541674 | 31.334221 | South African Wildlife College |
| C019 | 0.02 | | | 0.98 | | Y | | Hair | | AWC | | AWC | Unknown | 2010 | 2331CC | QDS | -23.876419 | 31.124565 | Phalaborwa town |
| C020 | 0.02 | | | 0.98 | | Y | | Hair | | AWC | | AWC | Unknown | Unknown | 2331CC | QDS | -23.876419 | 31.124565 | Phalaborwa town |
| C023 | 0.94 | | | 0.06 | | Y | | Hair | | DC | | DC | Unknown | 2011 | 2531AC | GPS | -25.390944 | 31.045540 | Second chance wildlife rehabilitation center |
| C024 | 0.96 | | | 0.04 | | Y | | Dry Tissue | | DC | | DC | DC | 2011 | 2331CC | GPS | -23.943068 | 31.166270 | Phalaborwa, Kruger National Park |
| C025 | 0.99 | | | 0.01 | | Y | | Dry Tissue | | DC | | DC | DC | 2011 | 2331CC | GPS | -23.943068 | 31.166270 | Phalaborwa, Kruger National Park |
| C027 | 0.96 | | | 0.04 | | Y | | Dry Tissue | | DC | | DC | AWC | 2010 | 2431DC | Error? | -24.876287 | 31.624592 | Skukuza museum, Kruger National Park (collection site unknown) |
| C028 | 0.01 | | | 0.99 | | Y | | Dry Tissue | | AWC | | AWC | AWC | 1987 | 2622AC | GPS | -26.433333 | 22.200000 | McGregor Museum, Kimberley |
| C029 | 0.04 | | | 0.96 | | Y | | Dry Tissue | | AWC | | AWC | AWC | Unknown | 2824DB | Error? | -28.625788 | 24.874166 | McGregor Museum, Kimberley |
| C030 | 0.01 | | | 0.99 | | Y | | Dry Tissue | | AWC | | AWC | AWC | 1987 | 2820AD | GPS | -28.450000 | 20.316667 | McGregor Museum, Kimberley |
| C031 | 0.01 | | | 0.99 | | Y | | Dry Tissue | | AWC | | AWC | AWC | 1987 | 2921DC | GPS | -29.866667 | 21.533333 | McGregor Museum, Kimberley |
| C032 | 0.01 | | | 0.99 | | Y | | Dry Tissue | | AWC | | AWC | AWC | 1988 | 2723CB | GPS | -27.700000 | 23.433333 | McGregor Museum, Kimberley |
| C033 | 0.02 | | | 0.98 | | Y | | Dry Tissue | | AWC | | AWC | AWC | 1987 | 2820AC | GPS | -28.466667 | 20.116667 | McGregor Museum, Kimberley |
| C034 | 0.02 | | | 0.99 | | Y | | Dry Tissue | | AWC | | AWC | AWC | 1987 | 2820AD | GPS | -28.450000 | 20.316667 | McGregor Museum, Kimberley |
| C035 | 0.17 | | | 0.84 | | Y | | Dry Tissue | | F1 BX AWC | | AWC | AWC | 1986 | 2620AB | GPS | -26.250000 | 20.316667 | McGregor Museum, Kimberley |
| C036 | 0.09 | | | 0.91 | | Y | | Dry Tissue | | AWC | | AWC | AWC | 1999 | 2918CA | GPS | -29.666667 | 18.000000 | McGregor Museum, Kimberley |
| C037 | 0.01 | | | 0.99 | | Y | | Dry Tissue | | AWC | | AWC | AWC | Unknown | 2824DB | Error? | -28.625788 | 24.874166 | McGregor Museum, Kimberley |
| C038 | 0.01 | | | 0.99 | | Y | | Dry Tissue | | AWC | | AWC | AWC | 1983 | 2624BD | GPS | -26.283333 | 24.966667 | McGregor Museum, Kimberley |
| C039 | 0.01 | | | 0.99 | | Y | | Dry Tissue | | AWC | | AWC | AWC | 1983 | 2723CB | GPS | -27.566667 | 23.366667 | McGregor Museum, Kimberley |
| C040 | 0.01 | | | 0.99 | | Y | | Dry Tissue | | AWC | | AWC | AWC | 1989 | 2522DD | GPS | -25.783333 | 22.933333 | McGregor Museum, Kimberley |
| C041 | 0.03 | | | 0.97 | | Y | | Dry Tissue | | AWC | | AWC | AWC | 1989 | 2522DD | GPS | -25.783333 | 22.933333 | McGregor Museum, Kimberley |
| C042 | 0.11 | | | 0.89 | | Y | | Dry Tissue | | F1 BX AWC | | AWC | Hybrid | 1987 | 2823DA | GPS | -28.716667 | 23.616667 | McGregor Museum, Kimberley |
| C043 | 0.99 | | | 0.01 | | Y | | Dry Tissue | | DC | | DC | Hybrid | 1983 | 2624BD | GPS | -26.283333 | 24.966667 | McGregor Museum, Kimberley |
| C044 | 0.12 | | | 0.88 | | Y | | Tissue | | F1 BX AWC | | AWC | AWC | Unknown | 2430BA | GPS | -24.218475 | 30.619926 | Hoedspruit |
| C045 | 0.01 | | | 0.99 | | Y | | Tissue | | AWC | | AWC | AWC | Unknown | 2430BD | QDS | -24.376349 | 30.874548 | Hoedspruit |
| C046 | 0.13 | | | 0.88 | | Y | | Tissue | | F1 BX AWC | | AWC | AWC | Unknown | 2430BD | QDS | -24.376349 | 30.874548 | Hoedspruit |
| C047 | 0.94 | | | 0.06 | | Y | | Tissue | | DC | | DC | DC | 2010 | 2431DC | GPS | -24.993460 | 31.601717 | Skukuza, Kruger National Park |
| C048 | 0.99 | | | 0.01 | | Y | | Tissue | | DC | | DC | DC | 2011 | 2431BD | GPS | -24.393950 | 31.777399 | Satara, Kruger National Park |
| C049 | 0.83 | | | 0.17 | | Y | | Tissue | | F1 BX DC | | DC | DC | 2010 | 2431CD | GPS | -24.978993 | 31.481724 | Kruger gate, Kruger National Park |
| C050 | 0.06 | | | 0.94 | | Y | | Tissue | | AWC | | AWC | AWC | 2006 | 2620BC | GPS | -26.470173 | 20.609870 | Kalahari Gemsbok National Park, Twee Rivieren rest camp |
| C051 | 0.01 | | | 0.99 | | Y | | Tissue | | AWC | | AWC | AWC | 2006 | 2620BC | GPS | -26.386194 | 20.698472 | Kalahari Gemsbok National Park |
| C052 | 0.91 | | | 0.09 | | Y | | Tissue | | DC | | DC | DC | 2006 | 2620BC | GPS | -26.470173 | 20.609870 | Kalahari Gemsbok National Park, Twee Rivieren rest camp |
| C055 | 0.01 | | | 0.99 | | Y | | Tissue | | AWC | | AWC | AWC | 2006 | 2620BC | GPS | -26.388140 | 20.701480 | Kalahari Gemsbok National Park |
| C056 | 0.01 | | | 0.99 | | Y | | Tissue | | AWC | | AWC | AWC | 2006 | 2620BC | GPS | -26.333844 | 20.744650 | Kalahari Gemsbok National Park |
| C057 | 0.02 | | | 0.98 | | Y | | Tissue | | AWC | | AWC | AWC | 2006 | 2620BC | GPS | -26.399359 | 20.690080 | Kalahari Gemsbok National Park |
| C058 | 0.01 | | | 0.99 | | Y | | Tissue | | AWC | | AWC | AWC | 2006 | 2620BC | GPS | -26.454194 | 20.600752 | Kalahari Gemsbok National Park |
| C059 | 0.13 | | | 0.87 | | Y | | Tissue | | F1 BX AWC | | AWC | AWC | 2006 | 2620BC | GPS | -26.327599 | 20.740160 | Kalahari Gemsbok National Park |
| C060 | 0.03 | | | 0.97 | | Y | | Tissue | | AWC | | AWC | AWC | 2006 | 2520CC | GPS | -25.762741 | 20.011098 | Kalahari Gemsbok National Park |
| C061 | 0.02 | | | 0.98 | | Y | | Tissue | | AWC | | AWC | AWC | 2006 | 2620BC | GPS | -26.365579 | 20.701190 | Kalahari Gemsbok National Park |
| C062 | 0.06 | | | 0.94 | | Y | | Tissue | | AWC | | AWC | AWC | 2006 | 2520CD | GPS | -25.974675 | 20.281200 | Kalahari Gemsbok National Park |
| C063 | 0.01 | | | 0.99 | | Y | | Tissue | | AWC | | AWC | AWC | 2006 | 2620BC | GPS | -26.370244 | 20.698270 | Kalahari Gemsbok National Park |
| C064 | 0.01 | | | 0.99 | | Y | | Tissue | | AWC | | AWC | AWC | 2006 | 2620BC | GPS | -26.389437 | 20.700120 | Kalahari Gemsbok National Park |
| C065 | 0.01 | | | 0.99 | | Y | | Tissue | | AWC | | AWC | AWC | 2006 | 2620BC | GPS | -26.388140 | 20.701480 | Kalahari Gemsbok National Park |
| C066 | 0.01 | | | 0.99 | | Y | | Tissue | | AWC | | AWC | AWC | 2006 | 2620BC | GPS | -26.415763 | 20.661910 | Kalahari Gemsbok National Park |
| C067 | 0.01 | | | 0.99 | | Y | | Tissue | | AWC | | AWC | AWC | 2006 | 2720BB | GPS | -27.105589 | 20.782356 | Between Molopo and Upington |
| C068 | 0.01 | | | 0.99 | | Y | | Tissue | | AWC | | AWC | AWC | 2006 | 2720BD | GPS | -27.285756 | 20.790253 | Between Molopo and Upington |
| C069 | 0.01 | | | 0.99 | | Y | | Tissue | | AWC | | AWC | AWC | 2006 | 2720BD | GPS | -27.446134 | 20.795059 | Between Molopo and Upington |
| C070 | 0.04 | | | 0.96 | | Y | | Tissue | | AWC | | AWC | AWC | 2006 | 2620DC | GPS | -26.912580 | 20.619621 | Between Molopo and Upington |
| C071 | 0.01 | | | 0.99 | | Y | | Tissue | | AWC | | AWC | AWC | 2006 | 2620BC | GPS | -26.354803 | 20.710270 | Kalahari Gemsbok National Park |
| C072 | 0.01 | | | 0.99 | | Y | | Tissue | | AWC | | AWC | AWC | 2006 | 2620BC | GPS | -26.397870 | 20.685050 | Kalahari Gemsbok National Park |
| C073 | 0.01 | | | 0.99 | | Y | | Tissue | | AWC | | AWC | AWC | 2006 | 2620BC | GPS | -26.376608 | 20.698770 | Kalahari Gemsbok National Park |
| C075 | 0.02 | | | 0.98 | | Y | | Tissue | | AWC | | AWC | AWC | 2006 | 2620BC | GPS | -26.390112 | 20.698820 | Kalahari Gemsbok National Park |
| C076 | 0.01 | | | 0.99 | | Y | | Tissue | | AWC | | AWC | AWC | 2006 | 2520CC | GPS | -25.811841 | 20.042630 | Kalahari Gemsbok National Park |
| C077 | 0.01 | | | 0.99 | | Y | | Tissue | | AWC | | AWC | AWC | 2006 | 2520CC | GPS | -25.873715 | 20.114310 | Kalahari Gemsbok National Park |
| C078 | 0.03 | | | 0.98 | | Y | | Tissue | | AWC | | AWC | AWC | 2006 | 2520CD | GPS | -25.968692 | 20.265690 | Kalahari Gemsbok National Park |
| C079 | 0.01 | | | 0.99 | | Y | | Tissue | | AWC | | AWC | AWC | 2006 | 2520CD | GPS | -25.995525 | 20.318190 | Kalahari Gemsbok National Park |
| C080 | 0.02 | | | 0.98 | | Y | | Tissue | | AWC | | AWC | AWC | 2006 | 2520CD | GPS | -25.995525 | 20.318190 | Kalahari Gemsbok National Park |
| C081 | 0.01 | | | 0.99 | | Y | | Tissue | | AWC | | AWC | AWC | 2006 | 2620BC | GPS | -26.403708 | 20.674150 | Kalahari Gemsbok National Park |
| C082 | 0.02 | | | 0.98 | | Y | | Tissue | | AWC | | AWC | AWC | 2006 | 2620BC | GPS | -26.397700 | 20.688270 | Kalahari Gemsbok National Park |
| C084 | 0.01 | | | 0.99 | | Y | | Tissue | | AWC | | AWC | AWC | 2006 | 2620BC | GPS | -26.409443 | 20.667070 | Kalahari Gemsbok National Park |
| C085 | 0.02 | | | 0.99 | | Y | | Tissue | | AWC | | AWC | AWC | 2006 | 2620BC | GPS | -26.398200 | 20.684280 | Kalahari Gemsbok National Park |
| C086 | 0.01 | | | 0.99 | | Y | | Tissue | | AWC | | AWC | AWC | 2006 | 2620BC | GPS | -26.398763 | 20.685150 | Kalahari Gemsbok National Park |
| C087 | 0.01 | | | 0.99 | | Y | | Tissue | | AWC | | AWC | AWC | 2006 | 2620BC | GPS | -26.271903 | 20.793750 | Kalahari Gemsbok National Park |
| C088 | 0.02 | | | 0.98 | | Y | | Tissue | | AWC | | AWC | Unknown | 2006 | 2620BC | GPS | -26.309299 | 20.750830 | Kalahari Gemsbok National Park |
| C089 | 0.01 | | | 0.99 | | Y | | Tissue | | AWC | | AWC | AWC | 2006 | 2620BC | GPS | -26.432045 | 20.626950 | Kalahari Gemsbok National Park |
| C090 | 0.01 | | | 0.99 | | Y | | Tissue | | AWC | | AWC | AWC | 2006 | 2620BA | GPS | -26.183730 | 20.592480 | Kalahari Gemsbok National Park |
| C091 | 0.01 | | | 0.99 | | Y | | Tissue | | AWC | | AWC | AWC | 2006 | 2620BC | GPS | -26.474397 | 20.610860 | Kalahari Gemsbok National Park |
| C092 | 0.99 | | | 0.01 | | Y | | Hair | | DC | | DC | DC | 2006 | 2520CC | GPS | -25.770214 | 20.001984 | Mata Mata rest camp, Kalahari Gemsbok National Park, |
| C093 | 0.99 | | | 0.02 | | Y | | Hair | | DC | | DC | DC | 2006 | 2520CC | QDS | -25.876095 | 20.123911 | Kalahari tented camp, Kalahari Gemsbok National Park, |
| C094 | 0.99 | | | 0.02 | | Y | | Hair | | DC | | DC | DC | 2006 | 2620BC | GPS | -26.470173 | 20.609870 | Twee Rivieren rest camp, Kalahari Gemsbok National Park, |
| C095 | 0.01 | | | 1.00 | | Y | | Dry Tissue | | AWC | | AWC | AWC | 2006 | 2620DC | QDS | -26.875973 | 20.623931 | Molopo Lodge |
| C096 | 0.99 | | | 0.01 | | Y | | Hair | | DC | | DC | DC | 2006 | 2520CC | QDS | -25.876095 | 20.123911 | Mata Mata rest camp, Kalahari Gemsbok National Park |
| C097 | 0.02 | | | 0.98 | | Y | | Hair | | AWC | | AWC | AWC | 2006 | 2821AC | QDS | -28.375794 | 21.123945 | Upington |
| C098 | 0.05 | | | 0.95 | | Y | | Hair | | AWC | | AWC | DC | 2006 | 2620DC | QDS | -26.875973 | 20.623931 | Andriesvale (60 km from Kgalagadi Transfrontier Park) |
| C099 | 0.00 | | | 0.00 | | N | | Hair | | n/a | | n/a | DC | 2006 | 2620DC | QDS | -26.875973 | 20.623931 | Andriesvale (60 km from Kgalagadi Transfrontier Park) |
| C101 | 0.99 | | | 0.01 | | Y | | Hair | | DC | | DC | DC | 2006 | 2620DC | QDS | -26.875973 | 20.623931 | Andriesvale (60 km from Kgalagadi Transfrontier Park) |
| C102 | 0.99 | | | 0.01 | | Y | | Hair | | DC | | DC | DC | 2006 | 2620DC | QDS | -26.875973 | 20.623931 | Andriesvale (60 km from Kgalagadi Transfrontier Park) |
| C103 | 0.99 | | | 0.01 | | Y | | Hair | | DC | | DC | DC | 2006 | 2620DC | QDS | -26.875973 | 20.623931 | Andriesvale (60 km from Kgalagadi Transfrontier Park) |
| C104 | 0.72 | | | 0.28 | | Y | | Hair | | F1 BX DC | | DC | DC | 2006 | 2620DC | QDS | -26.875973 | 20.623931 | Andriesvale (60 km from Kgalagadi Transfrontier Park) |
| C105 | 0.99 | | | 0.01 | | Y | | Hair | | DC | | DC | DC | 2006 | 2620DC | QDS | -26.875973 | 20.623931 | Andriesvale (60 km from Kgalagadi Transfrontier Park) |
| C106 | 0.99 | | | 0.01 | | Y | | Hair | | DC | | DC | DC | 2006 | 2620DC | QDS | -26.875973 | 20.623931 | Andriesvale (60 km from Kgalagadi Transfrontier Park) |
| C107 | 0.99 | | | 0.01 | | Y | | Hair | | DC | | DC | AWC | 2006 | 2620DC | QDS | -26.875973 | 20.623931 | Andriesvale (60 km from Kgalagadi Transfrontier Park) |
| C108 | 0.04 | | | 0.96 | | Y | | Dry Tissue | | AWC | | AWC | DC | 2006 | 2620DA | QDS | -26.626004 | 20.623933 | Welkom (10 km from Kgalagadi Transfrontier Park) |
| C109 | 0.98 | | | 0.02 | | Y | | Hair | | DC | | DC | DC | 2006 | 2620DA | QDS | -26.626004 | 20.623933 | Welkom (10 km from Kgalagadi Transfrontier Park) |
| C111 | 0.99 | | | 0.02 | | Y | | Hair | | DC | | DC | DC | 2006 | 2620DA | QDS | -26.626004 | 20.623933 | Welkom (10 km from Kgalagadi Transfrontier Park) |
| C112 | 0.99 | | | 0.01 | | Y | | Hair | | DC | | DC | DC | 2006 | 2620DA | QDS | -26.626004 | 20.623933 | Welkom (10 km from Kgalagadi Transfrontier Park) |
| C113 | 0.99 | | | 0.01 | | Y | | Hair | | DC | | DC | DC | 2006 | 2620DA | QDS | -26.626004 | 20.623933 | Welkom (10 km from Kgalagadi Transfrontier Park) |
| C114 | 0.97 | | | 0.03 | | Y | | Hair | | DC | | DC | DC | 2006 | 2620DA | QDS | -26.626004 | 20.623933 | Welkom (10 km from Kgalagadi Transfrontier Park) |
| C115 | 0.96 | | | 0.04 | | Y | | Hair | | DC | | DC | DC | 2006 | 2620DA | QDS | -26.626004 | 20.623933 | Welkom (10 km from Kgalagadi Transfrontier Park) |
| C116 | 0.96 | | | 0.04 | | Y | | Hair | | DC | | DC | DC | 2006 | 2620DA | QDS | -26.626004 | 20.623933 | Welkom (10 km from Kgalagadi Transfrontier Park) |
| C117 | 0.96 | | | 0.05 | | Y | | Hair | | DC | | DC | DC | 2006 | 2620DA | QDS | -26.626004 | 20.623933 | Welkom (10 km from Kgalagadi Transfrontier Park) |
| C118 | 0.99 | | | 0.02 | | Y | | Hair | | DC | | DC | DC | 2006 | 2620DA | QDS | -26.626004 | 20.623933 | Welkom (10 km from Kgalagadi Transfrontier Park) |
| C119 | 0.96 | | | 0.04 | | Y | | Hair | | DC | | DC | DC | 2006 | 2620DA | QDS | -26.626004 | 20.623933 | Welkom (10 km from Kgalagadi Transfrontier Park) |
| C120 | 0.99 | | | 0.01 | | Y | | Hair | | DC | | DC | DC | 2006 | 2620DA | QDS | -26.626004 | 20.623933 | Welkom (10 km from Kgalagadi Transfrontier Park) |
| C123 | 0.93 | | | 0.07 | | Y | | Hair | | DC | | DC | DC | 2006 | 2620DA | QDS | -26.626004 | 20.623933 | Welkom (10 km from Kgalagadi Transfrontier Park) |
| C124 | 0.96 | | | 0.05 | | Y | | Hair | | DC | | DC | DC | 2006 | 2620DA | QDS | -26.626004 | 20.623933 | Welkom (10 km from Kgalagadi Transfrontier Park) |
| C125 | 0.99 | | | 0.01 | | Y | | Hair | | DC | | DC | DC | 2006 | 2620DA | QDS | -26.626004 | 20.623933 | Welkom (10 km from Kgalagadi Transfrontier Park) |
| C126 | 0.01 | | | 0.99 | | Y | | Hair | | AWC | | AWC | DC | 2006 | 2620DC | QDS | -26.875973 | 20.623931 | Andriesvale (60 km from Kgalagadi Transfrontier Park) |
| C127 | 0.03 | | | 0.97 | | Y | | Tissue | | AWC | | AWC | AWC | 2006 | 2620DC | QDS | -26.875973 | 20.623931 | Andriesvale (60 km from Kgalagadi Transfrontier Park) |
| C129 | 0.01 | | | 0.99 | | Y | | Tissue | | AWC | | AWC | AWC | 2006 | 2620BA | GPS | -26.183730 | 20.592480 | Kalahari Gemsbok National Park |
| C130 | 0.80 | | | 0.20 | | Y | | Tissue | | F1 BX DC | | DC | Hybrid | 2006 | 2620BC | GPS | -26.470173 | 20.609870 | Kalahari Gemsbok National Park |
| C131 | 0.55 | | | 0.45 | | Y | | Tissue | | F1-2 HYB | | Hybrid | Hybrid | 2006 | 2620BC | GPS | -26.470173 | 20.609870 | Kalahari Gemsbok National Park |
| C132 | 0.44 | | | 0.56 | | Y | | Tissue | | F1-2 HYB | | Hybrid | Hybrid | 2006 | 2620BC | GPS | -26.470173 | 20.609870 | Kalahari Gemsbok National Park |
| C133 | 0.01 | | | 0.99 | | Y | | Tissue | | AWC | | AWC | AWC | 2006 | 2620DC | QDS | -26.875973 | 20.623931 | Andriesvale (60 km from Kgalagadi Transfrontier Park) |
| C135 | 0.01 | | | 0.99 | | Y | | Tissue | | AWC | | AWC | AWC | 2006 | 3225BA | QDS | -32.125414 | 25.624183 | Cradock |
| C136 | 0.01 | | | 0.99 | | Y | | Tissue | | AWC | | AWC | AWC | 2006 | 2921CC | QDS | -29.875620 | 21.123930 | Boomrivier |
| C137 | 0.02 | | | 0.98 | | Y | | Tissue | | AWC | | AWC | AWC | 2006 | 2921CC | QDS | -29.875620 | 21.123930 | Boomrivier |
| C138 | 0.02 | | | 0.98 | | Y | | Tissue | | AWC | | AWC | AWC | 2006 | 2921CC | QDS | -29.875620 | 21.123930 | Boomrivier |
| C139 | 0.01 | | | 0.99 | | Y | | Tissue | | AWC | | AWC | AWC | 2006 | 2921CC | QDS | -29.875620 | 21.123930 | Boomrivier |
| C140 | 0.72 | | | 0.28 | | Y | | Tissue | | F1 BX DC | | DC | AWC | 2006 | 2620BC | Error? | -26.376036 | 20.665102 | unknown |
| C141 | 0.01 | | | 0.99 | | Y | | Dry Tissue | | AWC | | AWC | AWC | 1972 | 3025AA | GPS | -30.125000 | 25.125000 | National Museum, Bloemfontein |
| C142 | 0.03 | | | 0.97 | | Y | | Dry Tissue | | AWC | | AWC | AWC | 1974 | 2927AA | GPS | -29.125000 | 27.125000 | National Museum, Bloemfontein |
| C146 | 0.13 | | | 0.88 | | Y | | Dry Tissue | | F1 BX AWC | | AWC | AWC | 1976 | 2924BB | GPS | -29.125000 | 24.875000 | National Museum, Bloemfontein |
| C149 | 0.02 | | | 0.99 | | Y | | Dry Tissue | | AWC | | AWC | AWC | 1978 | 2925DA | GPS | -29.625000 | 25.625000 | National Museum, Bloemfontein |
| C150 | 0.08 | | | 0.93 | | Y | | Dry Tissue | | AWC | | AWC | AWC | 1978 | 2927CC | GPS | -29.875000 | 27.125000 | National Museum, Bloemfontein |
| C151 | 0.24 | | | 0.77 | | Y | | Dry Tissue | | F1 BX AWC | | AWC | AWC | 1980 | 2829AA | GPS | -28.125000 | 29.125000 | National Museum, Bloemfontein |
| C152 | 0.08 | | | 0.92 | | Y | | Dry Tissue | | AWC | | AWC | AWC | 1982 | 2828BD | GPS | -28.375000 | 28.875000 | National Museum, Bloemfontein |
| C153 | 0.05 | | | 0.95 | | Y | | Dry Tissue | | AWC | | AWC | AWC | 1982 | 2828CA | GPS | -28.625000 | 28.125000 | National Museum, Bloemfontein |
| C154 | 0.06 | | | 0.94 | | Y | | Dry Tissue | | AWC | | AWC | AWC | 1982 | 2828CA | GPS | -28.625000 | 28.125000 | National Museum, Bloemfontein |
| C155 | 0.09 | | | 0.91 | | Y | | Dry Tissue | | AWC | | AWC | AWC | 1982 | 2828BB | GPS | -28.125000 | 28.875000 | National Museum, Bloemfontein |
| C156 | 0.01 | | | 0.99 | | Y | | Dry Tissue | | AWC | | AWC | AWC | 1982 | 2925CD | GPS | -29.875000 | 25.375000 | National Museum, Bloemfontein |
| C158 | 0.06 | | | 0.95 | | Y | | Dry Tissue | | AWC | | AWC | AWC | 1983 | 2725DA | GPS | -27.625000 | 25.625000 | National Museum, Bloemfontein |
| C159 | 0.14 | | | 0.86 | | Y | | Dry Tissue | | F1 BX AWC | | AWC | AWC | 1983 | 2927AC | GPS | -29.375000 | 27.125000 | National Museum, Bloemfontein |
| C160 | 0.06 | | | 0.94 | | Y | | Dry Tissue | | AWC | | AWC | AWC | 1984 | 2828CC | GPS | -28.875000 | 28.125000 | National Museum, Bloemfontein |
| C161 | 0.03 | | | 0.98 | | Y | | Dry Tissue | | AWC | | AWC | AWC | 1985 | 3027DC | GPS | -30.875000 | 27.625000 | National Museum, Bloemfontein |
| C162 | 0.03 | | | 0.97 | | Y | | Dry Tissue | | AWC | | AWC | AWC | 1985 | 3127AD | GPS | -31.375000 | 27.375000 | National Museum, Bloemfontein |
| C163 | 0.04 | | | 0.96 | | Y | | Dry Tissue | | AWC | | AWC | AWC | 1985 | 3128AC | GPS | -31.375000 | 28.125000 | National Museum, Bloemfontein |
| C164 | 0.01 | | | 0.99 | | Y | | Dry Tissue | | AWC | | AWC | AWC | 1986 | 3126BB | GPS | -31.125000 | 26.875000 | National Museum, Bloemfontein |
| C165 | 0.02 | | | 0.98 | | Y | | Dry Tissue | | AWC | | AWC | AWC | 1986 | 3127BB | GPS | -31.125000 | 27.875000 | National Museum, Bloemfontein |
| C166 | 0.04 | | | 0.96 | | Y | | Dry Tissue | | AWC | | AWC | AWC | 1986 | 3126DB | GPS | -31.625000 | 26.875000 | National Museum, Bloemfontein |
| C167 | 0.02 | | | 0.98 | | Y | | Dry Tissue | | AWC | | AWC | AWC | 1986 | 3028CA | GPS | -30.625000 | 28.125000 | National Museum, Bloemfontein |
| C168 | 0.95 | | | 0.05 | | Y | | Dry Tissue | | DC | | DC | AWC | 1987 | 3127DD | GPS | -31.875000 | 27.875000 | National Museum, Bloemfontein |
| C169 | 0.16 | | | 0.84 | | Y | | Dry Tissue | | F1 BX AWC | | AWC | AWC | 1987 | 3127DD | GPS | -31.875000 | 27.875000 | National Museum, Bloemfontein |
| C170 | 0.02 | | | 0.98 | | Y | | Dry Tissue | | AWC | | AWC | AWC | 1987 | 3127CB | GPS | -31.625000 | 27.375000 | National Museum, Bloemfontein |
| C171 | 0.08 | | | 0.92 | | Y | | Dry Tissue | | AWC | | AWC | AWC | 1998 | 2725DA | GPS | -27.625000 | 25.625000 | National Museum, Bloemfontein |
| C173 | 0.03 | | | 0.98 | | Y | | Dry Tissue | | AWC | | AWC | AWC | Unknown | 3327BB | GPS | -33.125000 | 27.875000 | Amathole Museum |
| C175 | 0.06 | | | 0.94 | | Y | | Dry Tissue | | AWC | | AWC | AWC | 1980 | 3320DB | GPS | -33.625000 | 20.875000 | Amathole Museum |
| C177 | 0.02 | | | 0.98 | | Y | | Dry Tissue | | AWC | | AWC | AWC | 1977 | 3320CC | GPS | -33.875000 | 20.125000 | Amathole Museum |
| C178 | 0.01 | | | 0.99 | | Y | | Dry Tissue | | AWC | | AWC | AWC | 1962 | 3226BD | GPS | -32.375000 | 26.875000 | Amathole Museum |
| C184 | 0.32 | | | 0.68 | | Y | | Dry Tissue | | F1 BX AWC | | AWC | AWC | 1963 | 3226BD | GPS | -32.375000 | 26.875000 | Amathole Museum |
| C185 | 0.06 | | | 0.94 | | Y | | Dry Tissue | | AWC | | AWC | AWC | 1978 | 2821CA | GPS | -28.625000 | 21.125000 | Amathole Museum |
| C186 | 0.06 | | | 0.94 | | Y | | Dry Tissue | | AWC | | AWC | AWC | 1966 | 3227DC | GPS | -32.875000 | 27.625000 | Amathole Museum |
|  |  | | |  | |  | |  | |  | |  |  |  |  |  |  |  |  |
|  | | | | |  |  |  |  |  |  |  |  |  |  |  |  |  |  |  |
|  | | |  | | | |  | | | |  |  |  |  |  |  |  |  |  |
|  | | | | |  |  |  |  |  |  |  |  |  |  |  |  |  |  |  |
|  | |  | | | | | | |  |  |  |  |  |  |  |  |  |  |  |

**Table S2: Details of microsatellite primers used in this study**

**________________________________________________________________________________________**

**Locus name Primer sequence (5’ – 3’)^*^ Fluorescent label Multiplex**

**________________________________________________________________________________________**

FCA220 CGATGGAAATTGTATCCATGG HEX 1

GAATGAAGGCAGTCACAAACTG 1

FCA097 TAATGTTCAACTTGAATTGCTTCC 6-FAM 1

GAACAGTAGTTTGCCCATACAGG 1

FCA005 CCTAAGGAAACAGTAATCCTGGC PET 1

TGGCAGGCATACCAGGAT 1

FCA678 AGCAATCTCCAGAATGTGTGG PET 1

TCAAAAGATTAAAGCCTTCCAA 1

FCA105 TTGACCCTCATACCTTCTTTGG NED 1

TGGGAGAATAAATTTGCAAAGC 1

FCA441 ATCGGTAGGTAGGTAGATATAG PET 2

GCTTGCTTCAAAATTTTCAC 2

FCA453 AATTCTGAGAACAAGCTGAGGG 6-FAM 2

ATCCTCTATGGCAGGACTTTG 2

FCA075 ATGCTAATCAGTGGCATTTGG 6-FAM 2

GAACAAAAATTCCAGACGTGC 2

________________________________________________________________________________________

**Table S2: continued**

**________________________________________________________________________________________**

**Locus name Primer sequence (5’ – 3’)^*^ Fluorescent label Multiplex**

**________________________________________________________________________________________**

FCA201 TCTGCAGGACCAGTCAGATG NED 2

AGCATACACAAATTGATGCTGG 2

FCA178 GTGCCCCATGAATCTCACTT NED 2

TACAACTCAGGGGTCGTATGG 2

FCA293 GATGGCCCAAAAGCACAC 6-FAM 3

CCCACATCTTGTCAACAACG 3

FCA310 TTAATTGTATCCCAAGTGGTCA 6-FAM 3

TAATGCTGCAATGTAGGGCA 3

FCA176 GGAAACTTGGAAAGCAAAACC PET 3

TCCACAGTTGGAGTTCTTAAGG 3

________________________________________________________________________________________

* For each locus the forward primer is listed followed by the reverse primer.

Table S3: Standard body measurements and mean annual home range for eight African wildcats observed during home range studies.

| **ID** | **Sex** | **Status** | **Body mass (kg)** | **TL** | **HB** | **T** | **Hf s/u** | **E** | **Months tracked** | **Hours** | **# fixes** | **Mean annual 100% MCP** | **Mean annual 95% MCP** | **Mean annual Core home range 50% kernel** |
| --- | --- | --- | --- | --- | --- | --- | --- | --- | --- | --- | --- | --- | --- | --- |
| VL01654 | ♀ | Adult | 4.5 | 90.0 | 59 | 31 | 14.5 | 6.2 | 31 | 547.9 | 3025 | 5.23 | 4 | 0.37 |
| VL01656 | ♀ | Adult | 4.0 | 98.0 | 64 | 34 | 14 | 7.5 | 26 | 206.7 | 1481 | 7.68 | 2.40 | 0.42 |
| VL01658 | ♀ | Adult | 4.6 | 96.0 | 62 | 34 | 15.7 | 7.7 | 6 | 72.5 | 516 | 5.85 | 4.19 | 0.24 |
| VL01662 | ♂ | Adult | 6.0 | 106.6 | 68 | 38.6 | 15.7 | 6.5 | 25 | 109.1 | 2625 | 12.31 | 10.74 | 0.40 |
| VL01665 | ♂ | Adult | 4.2 | 96.3 | 60.6 | 35.7 | 15.5 | 6.8 | 16 | 110.8 | 1912 | 12.71 | 7.82 | 1.09 |
| VL01667 | ♂ | Adult | 5.7 | 104.8 | 67 | 37.8 | 15.2 | 6.2 | 12 | 201.0 | 730 | 5.45 | 4.57 | 0.46 |
| VL01672 | ♂ | Adult | 6.0 | 100.6 | 63.8 | 36.8 | 16 | 7.5 | 6 | 100.0 | 579 | 8.67 | 7.80 | 0.76 |
| VL01673 | ♂ | Adult | 4.2 | 98.3 | 63.7 | 34.6 | 15.6 | 7.1 | 6 | 135.0 | 111 | 5.06 | 2.79 | 0.16 |

TL- Total length; HB- Head body length; T- Tail length; Hf s/u- Hind foot, E- Ear length measured in cm; Months tracked- number of months over which an individual cat was followed/observed; Hours- number of hours an individual cat was followed/observed; # fixes- number of GPS records taken; Mean annual 100% MCP- km^2^; Mean annual 95% MCP- km^2^; Mean annual Core home range 50% kernel- km^2^.


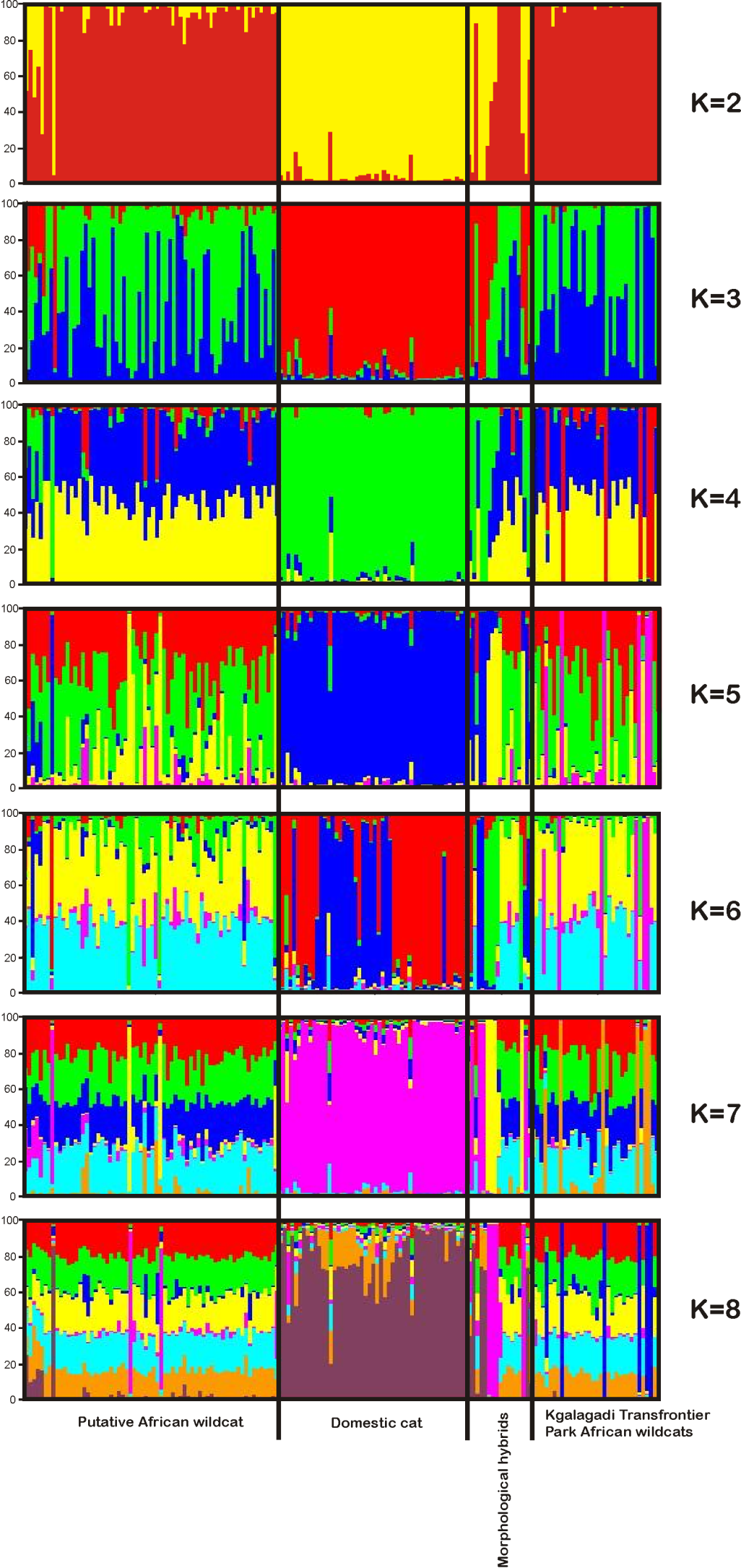


Figure S1: STRUCTURE bar plots where vertical axes illustrate the proportional assignment of individual genomes to the inferred genetic groups (*K* = 2 to *K*=8) for African wildcats outside protected areas, domestic cats, morphological hybrids, and individuals from the Kgalagadi Transfrontier Park. Membership of each individual’s genome to genetic clusters is indicated by different colours of vertical bars.
